# The human laryngeal microbiome: effects of cigarette smoke and reflux

**DOI:** 10.1038/srep35882

**Published:** 2016-10-24

**Authors:** Marie E. Jetté, Kimberly A. Dill-McFarland, Alissa S. Hanshew, Garret Suen, Susan L. Thibeault

**Affiliations:** 1 Department of Surgery, Division of Otolaryngology-Head and Neck Surgery, University of Wisconsin-Madison, Madison, Wisconsin, USA; 2 Department of Bacteriology, University of Wisconsin-Madison, Madison, Wisconsin, USA

## Abstract

Prolonged diffuse laryngeal inflammation from smoking and/or reflux is commonly diagnosed as chronic laryngitis and treated empirically with expensive drugs that have not proven effective. Shifts in microbiota have been associated with many inflammatory diseases, though little is known about how resident microbes may contribute to chronic laryngitis. We sought to characterize the core microbiota of disease-free human laryngeal tissue and to investigate shifts in microbial community membership associated with exposure to cigarette smoke and reflux. Using 454 pyrosequencing of the 16S rRNA gene, we compared bacterial communities of laryngeal tissue biopsies collected from 97 non-treatment-seeking volunteers based on reflux and smoking status. The core community was characterized by a highly abundant OTU within the family Comamonadaceae found in all laryngeal tissues. Smokers demonstrated less microbial diversity than nonsmokers, with differences in relative abundances of OTUs classified as *Streptococcus*, unclassified Comamonadaceae, *Cloacibacterium*, and *Helicobacter*. Reflux status did not affect microbial diversity nor community structure nor composition. Comparison of healthy laryngeal microbial communities to benign vocal fold disease samples revealed greater abundance of *Streptococcus* in benign vocal fold disease suggesting that mucosal dominance by *Streptococcus* may be a factor in disease etiology.

It has been estimated that 18 million Americans report voice problems each year^1^ and the associated short-term disability claims and work productivity losses are similar to asthma, heart disease, and depression^2^. Composed of stratified squamous epithelium and underlying lamina propria, vocal folds are located in the larynx at the junction between the respiratory and gastrointestinal tracts. Chronic laryngeal inflammation, which causes hoarseness, is commonly ascribed to reflux^3^, smoking^4^, allergies^5^, vocal overuse^6^ or a combination of these factors, and treatment is recommended empirically. Proton pump inhibitors (PPI) are prescribed to treat laryngeal inflammation; however, the use of PPI demonstrates limited efficacy^7^. In 2012 alone, more than 127 million US prescriptions and $9.5 billion dollars were spent on PPI, the most common medical management for chronic laryngeal inflammation^8^. An incomplete understanding of the pathophysiology of laryngeal inflammation is a major barrier to the development of improved medical therapies.

Given its exposure to inhaled, ingested, and refluxed microorganisms and irritants, it has been hypothesized that the larynx is an important organ for immunologic decision-making in the airway^9^. While there have been reports of the bacterial communities from the normal oral cavity^10^, nasal passages^11^, throat^10^ and lung^12^, there has been a paucity of research examining the microbiota of laryngeal tissue and its role in the pathophysiology of laryngeal disease. Four studies published to-date have examined the role of laryngeal microbes in disease, including chronic laryngitis^13^, vocal fold polyps^13^,^14^,^15^,^16^, nodules^14^, cysts^14^, laryngeal cancer^15^,^16^ and Reinke’s edema^14^; however, none have compared microbial data from disease to healthy laryngeal tissue. Moreover, no study has examined the contribution of two most commonly known mucosal irritants – cigarette smoke and reflux – to microbial community membership in the larynx.

There are multiple factors that may affect the local microbiota including temperature, anaerobiosis, pH, nutrients, host defenses and genetics, and antimicrobial agents^17^. Cigarette smoke contains thousands of chemical components including nitric oxide, carbon monoxide, nicotine, formaldehyde, acetone, ammonia, and acrolein, among many others^18^. These byproducts come in direct contact with the laryngeal mucosa and are associated with inflammation and erythema^18^,^19^. It has been suggested that components of tobacco smoke may have a selective toxic effect on specific microbes and that smoking enhances anaerobiosis in the oral cavity^20^. Further, persons who smoke often have an associated cough^21^ leading to mechanical tissue damage via repeated stress and strain of the laryngeal tissues. In this study, we hypothesized that smoking would result in decreased microbial diversity compared to a nonsmoking larynx, and an increased relative abundance of anaerobic taxa.

Refluxed material from the stomach includes gastric acid, pepsin, pancreatic enzymes, and bile acids^22^. The pH of the healthy, adult human stomach is low (1.5), a feature that may prevent gut colonization by foodborne pathogens^23^. The presence of a feeding nasogastric tube has been associated with a high prevalence of similar pathogenic microbes isolated from both the oropharynx and gastric contents, suggesting that refluxed material from the stomach may be the source of colonization of the upper respiratory tract^24^. We therefore predicted that the microbiota identified in the laryngeal tissue of participants with reflux would be similar to those found in the stomach.

To generate a more complete description of the microbiota that may contribute to laryngeal inflammation, we assessed the laryngeal microbiome of 97 non-treatment-seeking, healthy volunteers by sequencing the 16S rRNA gene using 454 pyrosequencing. Our goal was to identify microbial community shifts associated with smoking and reflux and to illuminate the presence of potential pathogens. To make a direct comparison between the healthy, non-treatment-seeking participants included in this study and treatment-seeking patients with documented laryngeal disease, we also analyzed our data in parallel with previously published data that investigated microbial communities in benign vocal fold lesions^14^.

## Results

### Participant Characteristics

Of 111 false vocal fold biopsies that yielded sufficient concentrations of DNA for downstream assays, 97 were successfully pyrosequenced. Pyrosequenced biopsies included those from 77 nonsmokers, of whom 19 could be classified as having gastroesophageal reflux disease (GERD) based on MII/pH^25^, 28 with laryngopharyngeal reflux (LPR)^25^, and 30 with no evidence of pathologic reflux (i.e., Normal). Of 20 smokers, 10 had GERD, 6 had LPR, and 4 were normal (Table 1). Mean and median age and gender of participants from whom biopsy tissue was pyrosequenced are detailed in Table 1 and additional details are outlined in Supplementary Table S1.

### Sequencing Results

**Figure 1:**
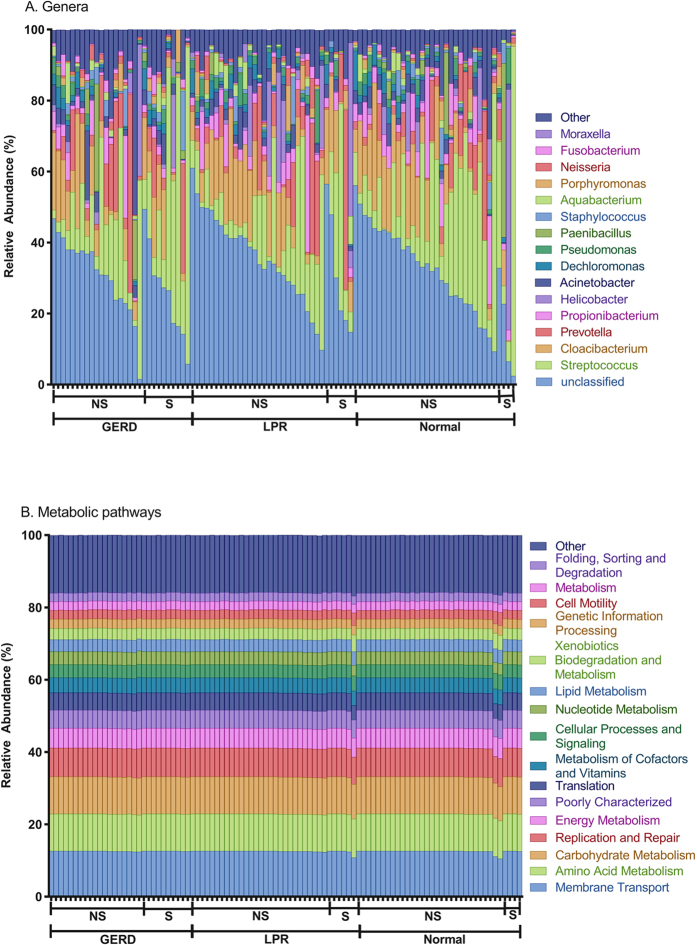
Relative abundance (%) of common genera (**A**) and KEGG Ortholog (**B**) across 97 participants.

Following bioinformatics processing, the total number of reads was 300,693, and the mean number of reads per sample was 3,100 (range = 341–21,715). Only samples with at least 340 sequences and a Good’s coverage >94% were included in further analyses (Supplementary Table S1). OTU analysis revealed 1347 total OTUs at 97% similarity across 97 samples. OTUs present at >1% relative abundance belonged to 5 phyla: Proteobacteria (mean 38% +/− standard deviation 20%), Firmicutes (34% +/− 21%), Bacteroidetes (19% +/− 10%), Actinobacteria (7% +/− 7%), and Fusobacteria (1% +/−2%). The 10 most abundant OTUs were classified as an unclassified genus of *Comamonadaceae* (26.5 +/− 13.1%), *Streptococcus* (19.9% +/−20.4%), *Cloacibacterium* (10% +/− 8.7%), *Prevotella* (6.9% +/− 10.9%), *Propionibacterium* (4.4% +/− 6.3%), *Helicobacter* (3.6% +/− 9.8%), *Veillonella* (2.8% +/− 4.3%), *Acinetobacter* (2.6% +/− 2.8%), *Pseudomonas* (1.8% +/− 2.2%), and *Bacillus* (1.8% +/− 2.2%; Fig. 1A; Supplementary Table S2).

### Richness, Diversity, and Coverage

After normalization, Chao1 richness estimates ranged from 19.2–181 across all samples (Supplementary Table S1). Bacterial diversity within samples varied between groups, with mean Shannon’s diversity indices ranging from 1.67–2.76 (Table 2). The inverse Simpson index ranged from 4.24 to 9.79. (Table 2; see Supplementary Table S1 for individual metrics).

Shannon diversity index differed in smokers compared to nonsmokers (ANOVA, TukeyHSD, p = 0.002; Supplementary Table S3), with smokers having decreased diversity relative to nonsmokers. Chao1 richness differed relative to reflux status, specifically participants with GERD demonstrated increased richness compared to those without reflux (i.e., Normal, p = 0.002).

**Figure 2:**
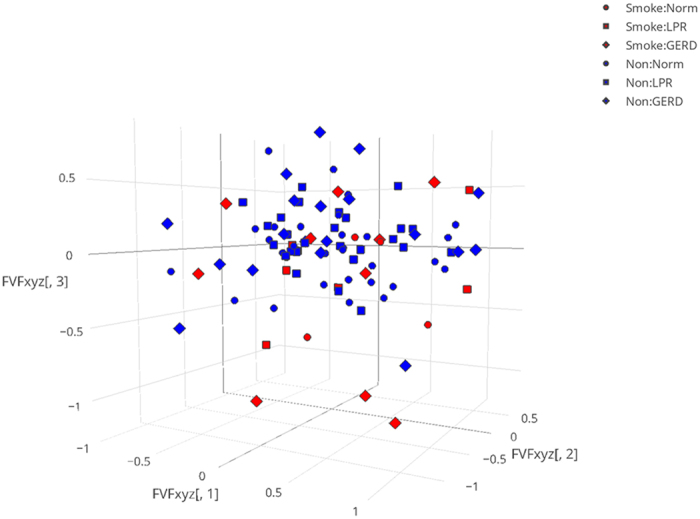
Comparison of bacterial community structure of false vocal fold biopsies. Nonmetric multidimensional scaling (nMDS) plot of the Bray-Curtis diversity index calculated from square root transformed OTU table. Lowest stress: 0.159. 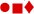 Smokers (Normal, LPR, GERD); 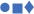 Nonsmokers (Normal, LPR, GERD).

Total community structure (Bray-Curtis) and composition (Jaccard) differed relative to smoking status (PERMANOVA, p = 0.028 and p = 0.026, respectively; Supplementary Table S4), but not relative to other variables including reflux status, age, and sex (p > 0.05), as visualized in the nMDS plot (Fig. 2). The effect of smoking was associated with differences in OTUs identified as *Streptococcus* (SIMPER, 17.6% contribution to difference), unclassified Commamonadaceae (11%), *Cloacibacterium* (6.8%), and others as outlined in Supplementary Table S5. A random variable included in all tests was not significant for any.

### PICRUSt/KEGG Analysis

While 16S rRNA analysis provides an indication of the bacteria present in a given sample, it does not provide information as to their function. To address this, we performed an analysis of our data using the program PICRUSt^26^, which indirectly infers function based on the known pathways of organisms categorized to a given species level OTU. Our analysis revealed a number of dominant KEGG pathways across all false vocal fold biopsies including membrane transport, amino acid metabolism, carbohydrate metabolism, replication and repair, and energy metabolism (Fig. 1B). Supplementary Table S6 outlines predicted relative abundances of Level 1, 2, and 3 KEGG pathways based on sequenced relatives generated using PICRUSt.

### Comparison of False Vocal Fold to Benign Vocal Fold Lesion Communities

To identify differences in the microbiota in healthy laryngeal tissue relative to diseased, data were analyzed in parallel with data collected from patients with documented vocal fold lesions (N = 44^14^). The total number of false vocal fold biopsy and vocal fold lesion sequencing reads was 524,570 and after normalization these contained 741 OTUs (Supplementary Table S2).

**Figure 3:**
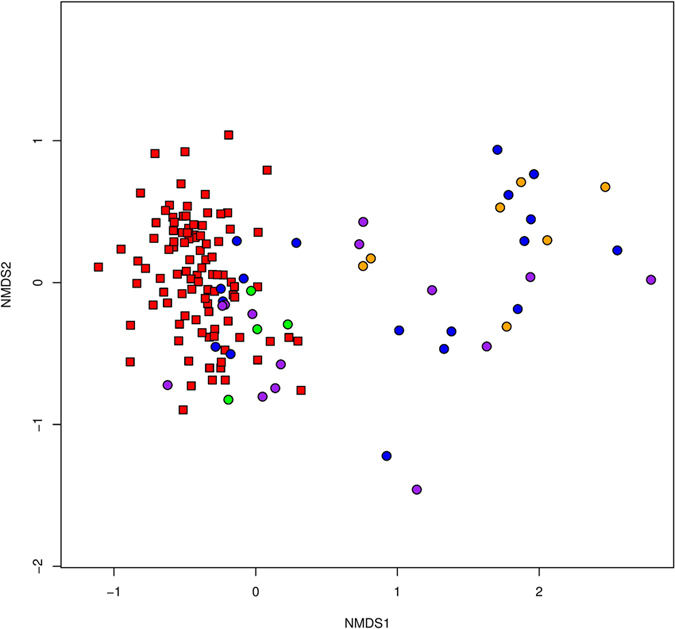
Comparison of bacterial community structure of false vocal fold biopsies and vocal fold lesions. Nonmetric multidimensional scaling (nMDS) plot of the Bray-Curtis diversity index calculated from square root transformed OTU table. Lowest stress: 0.159. 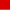 False Vocal Fold Biopsies; 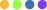 Vocal Fold Lesions (Reinke’s edema, cyst, nodule, polyp).

**Figure 4:**
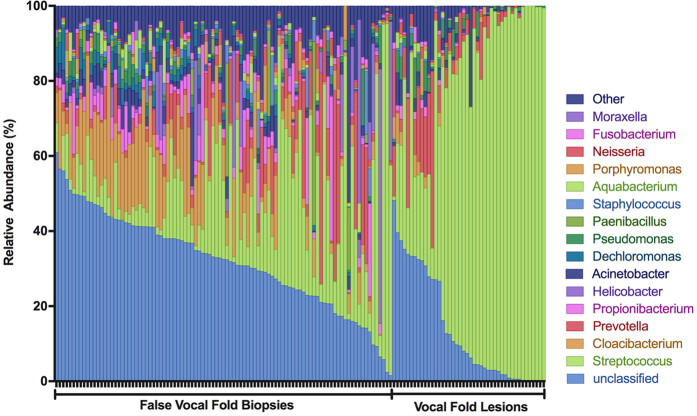
Relative abundance of common genera across all false vocal fold biopsies and vocal fold lesions.

**Figure 5:**
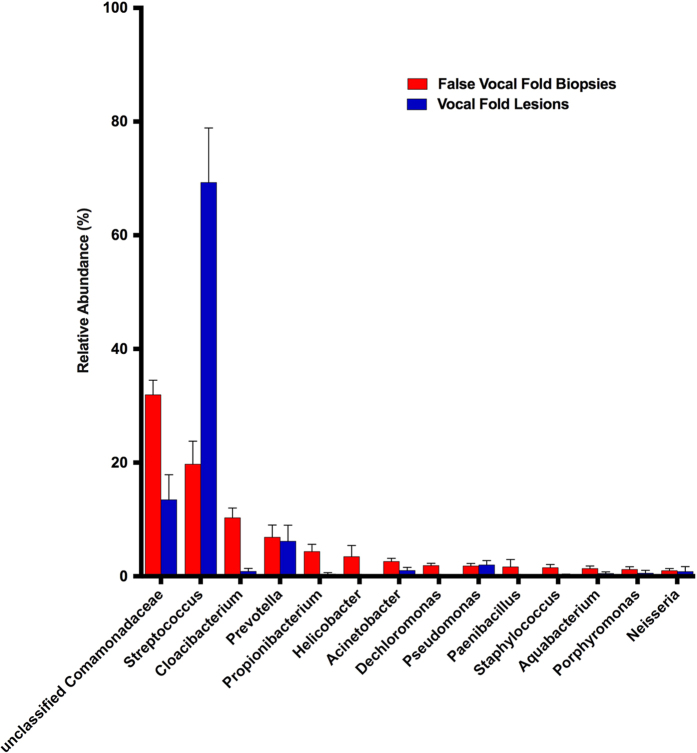
Relative abundance of top genera found in false vocal fold biopsies and vocal fold lesions. Error bars represent 95% confidence intervals.

False vocal fold biopsies were more rich and diverse than vocal fold lesions by Chao1 (ANOVA, TukeyHSD, p < 0.0001), inverse Simpson (p = 0.001) and Shannon metrics (p < 0.0001; Table 3). Specifically, healthy laryngeal tissue was more rich and diverse than polyps and Reinke’s edema (see Supplementary Table S7 for p-values). Total community structure (Bray-Curtis) and composition (Jaccard) also differed between false vocal fold biopsies and lesions (PERMANOVA, both p = 0.0001; Supplementary Table S8), as visualized in the nMDS plot (Fig. 3). In the nMDS plot, communities from false vocal fold biopsies and vocal fold lesions clustered together with some overlap between the two communities. Figures 4 and 5 demonstrate the preponderance of an unclassified OTU within the family Comamonadaceae and one within the genus Streptococcus across false vocal fold biopsies and vocal fold lesions, respectively.

## Discussion

While it has yet to be conclusively proven that individuals or even body sites harbor a “core” set of specific bacterial taxa^27^, the publication of multiple studies examining the laryngeal microbiota associated with disease in the past three years^14^,^15^,^16^, along with the data from healthy volunteers presented herein, has yielded a vastly improved characterization of the microbial composition of laryngeal tissue. Proposed core members are likely to adjust as technology evolves and sampling depth increases, distinguishing taxa that are absent from those that are merely rare.

The baseline bacterial community of the larynx regardless of smoking or reflux status contained a highly abundant OTU within the family Comamonadaceae. Some of the predominant genera are the same as those previously found in laryngeal disease including *Streptococcus* and *Prevotella*^14^,^15^,^16^, whereas *unclassified* Comamonadaceae and *Cloacibacterium* are additions to the existing laryngeal microbiota literature.

**Figure 6:**
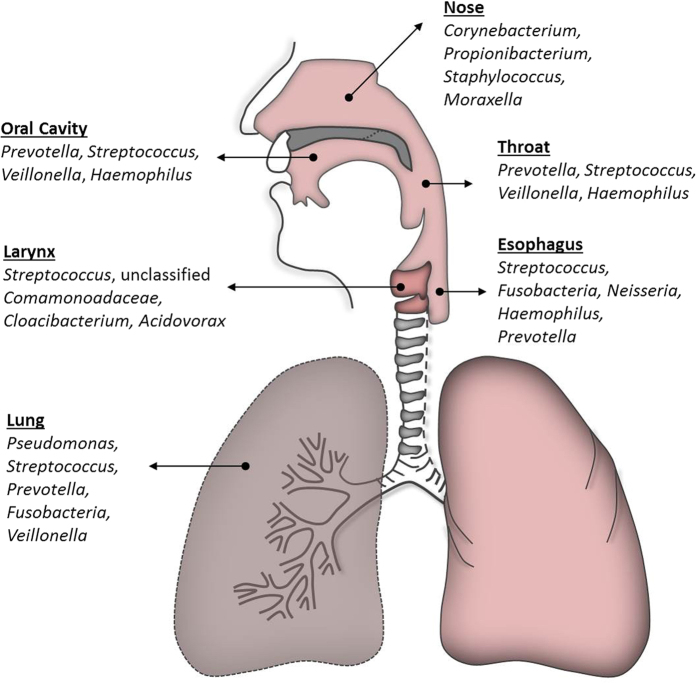
Schematic outlining common microbes across the aerodigestive tract^10^,^11^,^12^,^28^,^29^,^30^,^50^,^62^.

Of the common genera found in laryngeal tissue biopsies, several are known commensals or pathogens and many are also found in mucosal sites adjacent to the larynx, including the esophagus, lung, and mouth. One of the most abundant genera across all laryngeal biopsies in our study was *Streptococcus*. *Streptococcus* is a Gram positive genus of the phylum Firmicutes, of which there are over 50 species. Different species of *Streptococcus* have been implicated in upper respiratory tract infections, pharyngitis, pneumonia, otitis media, and sepsis. Multiple studies investigating the microbiota of the normal esophagus in both children^28^ and adults^29^ also revealed a predominance of *Streptococcus*. *Streptococcus* is similarly highly abundant in the healthy salivary microbiome^30^ and in the lung^12^ and oral cavity (including buccal mucosa, gingiva, palate, tongue, and oropharynx^10^; Fig. 6).

One taxon of interest from our nontreatment-seeking laryngeal dataset are bacteria in the family *Comamonadaceae*, a group of Gram negative aerobic *Proteobacteria*. *Comamonadaceae* has been found in the upper and lower airways, though at low abundance (<1%) compared to our findings^31^. Comparison of nasopharyngeal swabs from infants with acute otitis media and healthy controls revealed a greater abundance (4.8%) of *Comamonadaceae* in controls^31^, supporting the notion that this taxon is associated with a healthy respiratory tract. While human infections caused by members of *Comamonadaceae* are rare, there have been cases of associated infections reported in the literature^32^.

Within the disease-free laryngeal microbiome, there are a number of pathways that are present and abundant, such as those involved in membrane transport, replication and repair, and nucleotide metabolism. The ATP-binding cassette (ABC) transporter pathway, for example, was highly abundant across samples. Bacteria use ABC transporters to take up nutrients such as iron, peptides or sugars, and pump toxic components out of the cell^33^, and this may be the paradigm by which the laryngeal microbiota synthesize carbohydrates. The pathway for purine metabolism was also abundant across laryngeal samples, a finding that replicates data characterizing two distinct lung microbiomes^34^. Specifically, in the oral-bacteria predominant lung microbiome (i.e., pneumotype_SCT_^35^ or pneumotype_SPT_^34^), the pathway for purine metabolism was more abundant than in the lung microbiome characterized by background predominant taxa (i.e., pneumotype_UN_^35^ or pneumotype_BPT_^34^).

There were notable differences in community membership relative to smoking status. As predicted, smokers demonstrated reduced microbial diversity in this study. Further, smokers had bacterial communities with greater abundances of *Streptococcus* than nonsmokers. Many species of *Streptococcus* are facultative anaerobes, supporting our hypothesis that the microbiota of smokers would be dominated by anaerobes. Charlson *et al*.^36^ also found increases in anaerobic *Streptococcus* and *Veillonella* in the oropharynx of smokers.

In contrast to our prediction that the laryngeal microbiota of persons with reflux would be similar to the stomach microbiota, we did not find any shifts in microbial abundance associated with the effect of reflux status alone, though others have demonstrated reflux-related shifts. Rosen *et al*.^37^ investigated differences in gastric, lung, and oropharyngeal microbiota in children who underwent bronchoscopy and upper endoscopy for the evaluation of chronic cough. Patients taking PPI to treat reflux within the 24 hours prior to endoscopy were found to have increased relative abundance of *Streptococcus* in gastric fluid and oropharyngeal (i.e. posterior tongue) swabs, as well as elevated *Cloacibacterium* in the oropharynx. In patients who underwent reflux testing with combined multichannel intraluminal impedance pH monitoring that resulted in abnormal findings (i.e., pH <4 for >6% of the study time and greater than 73 reflux episodes), the authors observed greater abundance of oropharyngeal *Neisseria*, *Allobaculum*, *Alloiococcus*, *Cryseobacterium*, *Fusobacterium*, *Paenibacillus*, *Propionibacterium*, *Sphingobacterim*, and *Sphingomonas*. In the esophagus, increased relative abundance of *Streptococcus* has been observed in histologically normal tissue compared to tissue from patients with esophagitis^38^.

In our study, we found that *Helicobacter* was present at >1% relative abundance in 24/97 (25%) samples analyzed, and was detected in 45/97 (46%) biopsies. This finding is similar to epidemiologic data that suggest *H*. *pylori* infection in 52% of healthy asymptomatic volunteers^39^; however this differs from 454 data collected from benign vocal fold lesions in which only 5/44 lesions (11%) yielded sequences identified as *Helicobacter*^14^, and at very low abundances (1% or less). Research demonstrates that *H*. *pylori* prevents allergic airway inflammation and hyperresponsiveness in clinical models via immunomodulatory properties mediated by induction of T regulatory cells^40^. Given the Unified Airway Disease theory^41^, it is possible that the presence of *Helicobacter* in the tissue of healthy, non-treatment seeking volunteers included in this study confers protection from laryngeal disease, and the relative absence and low abundance of *Helicobacter* in benign vocal fold lesions is associated with the presence of disease. Participants were not excluded from either study based on antibiotic use; however, patients included in the study of benign vocal fold lesions were perhaps more likely to have been treated with antibiotics for a sore throat prior to surgical intervention for their disease. In a retrospective analysis, Linder & Stafford^42^ found that more than half of adults who present to their primary care physician with a principal complaint of sore throat were treated with antibiotics. Similarly, PPI use, which has been shown to affect *H*. *pylori* colonization in the colon^43^, is common in patients undergoing surgery for benign vocal fold lesions. Ultimately, PPI and antibiotic use in patients with vocal fold lesions could have resulted in the differences in *Helicobacter* abundance and prevalence observed in this group, unrelated to the lesion itself, relative to that observed in the healthy non-treatment seeking participants included in the present study. Results presented herein fail to provide additional scientific data to support the theory that laryngeal colonization with *Helicobacter* is associated with benign vocal fold disease.

This study is the third to examine microbial communities in the human larynx using next-generation sequencing. Hanshew, Jetté, and Thibeault^14^ first characterized the microbiota of benign vocal fold lesions, finding an abundance of *Streptococcus*, particularly *Streptococcus pseudopneumoniae* across all lesion types. The most striking difference between that study and the data described herein was the presence and abundance of *Streptococcus*, with lesions on average demonstrating 69.3% mean relative abundance of this genus compared to 19.7% mean relative abundance found in false vocal fold biopsies. Gong *et al*.^15^ used vocal fold polyps as control tissue in an investigation of the microbiota of laryngeal cancer and found 56% relative abundance of *Streptococcus*, which is more similar to the results from Hanshew *et al*.^14^ than this study. Taken together, these studies suggest an association between microbial communities dominated by *Streptococcus* and vocal fold pathology. It is also possible that the vocal fold mucosa differs from false vocal fold mucosa creating a niche for microbial communities dominated by *Streptococcus*, whereas the architecture of the false vocal fold mucosa allows for greater diversity. PPI use has been associated with increased relative abundance of gastrointestinal *Streptococcus* as measured from stool^44^. Given the propensity for treating voice problems with PPI^45^, it is possible that the increased abundance of *Streptococcus* found in vocal fold lesions relative to false vocal fold tissue relates to PPI use.

Studies of the laryngeal microbiome face unique challenges not present in body sites that are easily accessible or that contain high bacterial biomass such as skin, gut, oral cavity or genital tract. Sampling by laryngoscopy required passage through the upper respiratory tract, which harbors large microbial populations where contaminating organisms could have been acquired. In addition, the miniscule false vocal fold biopsies collected in this study were of low microbial biomass, meaning that low-level contamination of sequences from dust, reagents, instruments, or other sources may have confounded the data. Further, though processed in the same facilities using the same reagents, reliability of data comparison between low biomass false vocal fold tissue to low biomass vocal fold lesions is limited by potential batch effects as these tissues were collected, processed, and sequenced on different days by different experimenters.

While the data set used in this study was well-characterized relative to variables of interest (inclusion criteria along with reflux and smoking status), we did not measure all of the biological or environmental variables that could have contributed to variation in bacterial communities. Demographic features that have been documented in the literature as being associated with shifts in bacterial communities include body mass index^46^, diet^47^, hormone levels^48^, and disease states^49^. Similarly, there are temporal shifts in local microbiota based on the factors described above, particularly in the oral mucosa^50^ that our data cannot capture.

We conclude that the diversity of the laryngeal microbiome is affected by smoking, but not by reflux. As hypothesized, smoking specifically contributed to differences in abundance of *Streptococcus*, an anaerobic bacterium, across laryngeal biopsies. *Streptococcus* was also found in greater abundance in benign vocal fold lesions compared with laryngeal tissue biopsies taken from disease-free non-treatment-seeking participants. Taken together, these findings suggest that a preponderance of *Streptococcus*, possibly due to tissue characteristics altered by cigarette smoke, may be a factor in laryngeal disease etiology. Future studies investigating temporal shifts in laryngeal microbial populations in healthy and diseased participants taking into account considerations like diet, body mass index, and antibiotic and PPI use, and limiting batch effects, are certainly warranted.

## Methods

### Participant Selection

Participants aged 21–65 years were recruited with newspaper and email advertisements and signs in the clinic and around the University of Wisconsin-Madison and Madison, WI. Participants underwent video laryngostroboscopic examination and 24-hour MII/pH, with each procedure performed on separate dates. The protocol was approved by the Institutional Review Board of University of Wisconsin-Madison, informed consent was obtained from all participants, and all experiments were performed in accordance with relevant institutional guidelines and regulations. Participants were excluded from the study if they had a history of radiation therapy to the head and neck within the past five years, lung or gastroesophageal surgery, chronic sinusitis or rhinitis in the last year, an acute traumatic event near the larynx in the last year, tracheostomy or other significant laryngeal or tracheal surgery, and substance or alcohol abuse in the past year. Consumption of more than 10 (women) and 17 (men) units of alcohol per week (means of United Kingdom and United States recommended weekly limits) excluded participants^51^. Further exclusion criteria included malignancy (except superficial basal cell carcinoma) within the past five years, presence of an infectious cause of laryngitis in the past three months, need for continuous therapy with diazepam, phenytoin, mephenytoin, warfarin, anticholinergics, antineoplastics, prostaglandin analogs, histamine receptor (H2) antagonists, steroids (inhaled, oral or intravenous), promotility drugs and sucralfate, use of any PPI or H2 blockers in the past year, theophylline or any other investigational compound or participation in an investigational drug study in the previous 60 days. Women were excluded if pregnant or lactating. Nonsmokers had not smoked during the previous year. Smokers were defined by consumption of a minimum of 5 cigarettes/5 g of tobacco per day for the duration of one or more years, thereby distinguishing them from light smokers^52^,^53^.

### General Procedure

Participation involved three clinical visits on three separate days over the course of approximately three months. The first visit involved obtaining informed consent and performing video laryngostroboscopy at the Voice and Swallow Clinic within the University of Wisconsin (UW) Hospital to confirm absence of laryngeal disease. The second visit took place in the outpatient Gastroenterology Clinic at the UW Hospital wherein the multichannel intraluminal impedance with pH (MII/pH) catheter was placed. During the third visit, participants returned to the Voice and Swallowing Clinic and underwent transnasal laryngeal biopsy under local anesthetic.

### Sample Collection

Bacterial communities were sampled from false vocal fold tissue of non-treatment-seeking, healthy volunteers. An Olympus ENF-T3 flexible fiberoptic laryngoscope with biopsy forceps passed through a biopsy channel was used following topical anesthesia with 4% lidocaine. In all cases, sampling was completed aseptically. Tissue biopsies were snap frozen within seconds of retrieval and stored at −80 °C prior to molecular analysis.

### Genomic DNA Isolation

DNA was extracted with the EpiCenter MasterPure Complete DNA and RNA Purification Kit (Illumina, Madison, WI) with modifications to the manufacturer’s protocol. Samples were gently thawed at room temperature and briefly centrifuged to collect tissue. 300 μl of Tissue and Cell Lysis solution was added to the tube with the tissue. Lysis solution and tissue were then transferred to a sterile screw top tube containing 150–200 mg of 400 μM silica beads. 100 μg of proteinase K was added, tubes were vortexed, and incubated at 55 °C for 1 hour, with vortexing every 15 minutes. Bead tubes were then shaken in a horizontal adapter on the vortex for 10 minutes. 5 μg of RNase A was added, tubes were vortexed, and incubated at 37 °C for 30 min. The remainder of the manufacturer’s protocol was followed as written. DNA was resuspended in TE, quantified using a spectrophotometer (Nanodrop) and stored at −20 °C until use.

### Library Preparation

Barcoded PCRs were performed in triplicates containing 150 ng of template genomic DNA, 0.2 μl AccuPrime Taq DNA Polymerase (Life Technologies, Grand Island, NY), 2.5 μl Buffer II, 400 nM both forward and reverse primers, and water to 50 μl total. Thermocycling conditions were as follows: 95 °C 2 min, followed by 30 cycles of: 95 °C 20 sec, 56 °C 30 sec, 72 °C 1 min, and a final extension of 72 °C 8 min. Primers included 357F and 926R, as suggested by the Human Microbiome Project (HMP)^54^, where 357F contained the B adapter for 454 pyrosequencing, and 926R contained both the A adapter and a 10 base pair multiplex identifier. Triplicate PCRs were pooled and gel extracted from a low-melt agarose gel using Zymoclean Gel DNA Recovery Kit (Zymo Research, Irvine, CA) by visualizing on a blue light transilluminator (Clare Chemical Research, Dolores, CO). Cleaned PCR products were quantified using a Qubit fluorometer (Invitrogen, Grand Island, NY). Products were diluted and pooled at equal concentrations for 454.

### Roche 454 Pyrosequencing

454 pyrosequencing was conducted on a Roche GS Junior (Roche, Indianapolis, IN) using titanium chemistry and long read modifications found in Hanshew *et al*.^55^. Samples were sequenced across nine picotiter plates. Emulsion PCR and sequencing were done according to manufacturer’s protocols using the Lib-L kit with an initial emPCR ratio of one molecule of DNA per bead.

### Data Analysis

Raw data was processed using mothur (v. 1.37^56^ using the Standard Operating Procedure for 454 data (www.mothur.org/wiki/454_SOP, accessed June 24, 2016). Sequences were aligned to a Silva-derived reference database^57^. Chimeras were detected using UCHIME and removed and sequences were assigned to taxonomic groups using the GreenGenes database^58^. All eukaryotic, archaeal, and unclassifiable reads were removed after classify.seqs and sequences were assigned to operational taxonomic units (OTUs) at 97% sequence identity. Good’s coverage and OTU counts were calculated in mothur and samples with sufficient coverage (>94%) were then normalized to 340 sequences per sample. Mean relative abundance was calculated for each taxon across all participants and figures were generated in GraphPad Prism 7 (LaJolla, CA). OTU counts, Chao1, inverse Simpson’s Diversity, and Shannon’s Evenness were computed from normalized data using mothur.

### Statistics

All statistical analyses were performed using the vegan package^59^ in R^60^. Total microbial community structure (diversity, Bray-Curtis) and composition (richness, Jaccard) were calculated from square root transformed OTU data and visualized by non-metric multidimensional scaling (nMDS) plots. Community structure and composition were assessed for differences by permutational analysis of variance (PERMANOVA) at the OTU- and genus-levels. Community diversity (Shannon’s and Simpson’s) and richness (Chao) were assessed using ANOVA with Tukey’s HSD correction for multiple comparisons. For all tests, smoking status, reflux status, smoking:reflux, age, and sex were included as variables (Supplementary Table S2). Similarity percentage analysis (SIMPER) was used to identify the OTUs and genera that most contributed to the dissimilarity between smokers and nonsmokers observed in PERMANOVA. A p-value of p < 0.05 was considered statistically significant, and a random variable was included in all tests.

### Comparison with Vocal Fold Lesions

Data from 44 benign vocal fold lesions^14^ were included in further analysis to compare diseased laryngeal tissue to healthy (NCBI sequence read archive, SRP047304). Analyses were computed as described above, including Bray-Curtis, Jaccard, Good’s coverage, Chao, inverse Simpson, Shannon, PERMANOVA, and ANOVA with Tukey HSD. PERMANOVA tests between healthy tissue and different lesion types (nodules, polyps, cysts, and Reinke’s edema) were corrected for multiple comparisons with Bonferroni’s correction.

### Microbial Function Prediction

Microbial function was predicted using a software package designed to infer functional content from 16S rRNA data known as PICRUSt^26^. OTUs were mapped to Greengenes taxonomy^61^ at 97% similarity. Predicted genes and their function were normalized and aligned to Kyoto Encyclopedia of Genes and Genomes (KEGG) database.

## Additional Information

**Accession codes:** The data sets supporting the results of this article are available in the National Center for Biotechnology Information’s Sequence Read Archive Knowledge Base, PRJNA289913 and PRJNA260304.

**How to cite this article**: Jetté, M. E. *et al*. The human laryngeal microbiome: effects of cigarette smoke and reflux. *Sci. Rep.* **6**, 35882; doi: 10.1038/srep35882 (2016).

## Supplementary Material

Supplementary Information

Supplementary Dataset 1

## Figures and Tables

**Table 1:** Participant Characteristics.

	N	Median Age in Years	Mean Age in Years (range)	Female
*Nonsmoker*				
GERD	19	50	44 (21–65)	58%
LPR	28	32	36 (21–61)	54%
Normal	30	49.5	45 (23–65)	53%
*Smoker*				
GERD	10	49.5	46 (25–61)	60%
LPR	6	31.5	34 (26–49)	50%
Normal	4	41	44 (32–60)	50%
Total	97	42	42 (21–65)	56%

**Table 2:** Mean Sequence Richness, Evenness, and Diversity in False Vocal Fold Biopsies (±SEM).

	Chao1	1/Simpson	Shannon	Good’s coverage
*Nonsmoker*				
GERD	98.90 ± 10.23	9.79 ± 1.06	2.76 ± 0.12	0.97 ± 0.00
LPR	81.97 ± 5.17	7.96 ± 0.58	2.64 ± 0.06	0.98 ± 0.00
Normal	70.97 ± 4.64	8.02 ± 0.58	2.58 ± 0.07	0.98 ± 0.00
*Smoker*				
GERD	90.12 ± 16.68	8.05 ± 1.64	2.40 ± 0.29	0.98 ± 0.00
LPR	58.80 ± 8.35	6.35 ± 1.74	2.33 ± 0.21	0.99 ± 0.00
Normal	46.08 ± 13.24	4.24 ± 2.00	1.67 ± 0.52	0.99 ± 0.00
*All Samples*	79.81 ± 3.61	8.09 ± 0.39	2.56 ± 0.06	0.98 ± 0.00

**Table 3:** Mean Sequence Richness, Evenness, and Diversity in False Vocal Fold Biopsies and Vocal Fold Lesions (±SEM).

	Chao1	1/Simpson	Shannon	Good’s coverage
*False Vocal Fold Biopsies*	79.81 ± 3.61	8.09 ± 0.39	2.56 ± 0.06	0.9800 ± 0.00
*Vocal Fold Lesions*	39.27 ± 4.61	5.18 ± 0.89	1.55 ± 0.16	0.99 ± 0.00
