# Supplementary material for: The human laryngeal microbiome: effects of cigarette smoke and reflux
Source: Sci Rep. 2016 Oct 24;6:35882. doi: 10.1038/srep35882 (PMC5075886; doi:10.1038/srep35882)
Supplement: Supplementary Information [file srep35882-s1.pdf]

Additional Files

The human laryngeal microbiome: effects of cigarette smoke and reflux

Marie E. Jetté, Kimberly A. Dill-McFarland, Alissa S. Hanshew, Garret Suen, Susan L. Thibeault

Table S1. Detailed Participant Characteristics

Cohort	Age	Gender	# of sequences (normalized*)	Observed # of species (normalized)	Chao 1 (richness)	Good's coverage	1/Simpson (diversity)	Shannon (diversity)
**Nonsmoker**								
GERD	21	Male	2418 (337)	67 (33)	42.166667	0.991729	6.758505	2.444602
	22	Female	737 (321)	91 (51)	101.142857	0.945726	8.545757	2.786825
	23	Male	578 (350)	66 (66)	106.071429	0.956747	9.82229	3.031151
	25	Female	2555 (333)	89 (49)	92.875	0.991781	6.572015	2.577513
	31	Female	5595 (333)	109 (50)	83.333333	0.996604	9.039738	2.855619
	33	Female	1002 (326)	128 (70)	167.5	0.942116	13.035187	3.234239
	39	Male	1003 (329)	101 (61)	86	0.96012	12.917405	3.190083
	40	Female	775 (325)	98 (62)	117.111111	0.953548	18.38338	3.315683
	42	Male	1502 (332)	81 (50)	69.25	0.984021	10.262607	2.882488
	50	Female	5623(329)	136 (41)	58.142857	0.99342	6.493682	2.578841
	50	Male	1008 (327)	121 (63)	90.1875	0.94246	9.521436	3.029905
	52	Male	18638 (334)	134 (15)	19.2	0.996244	2.237597	1.052772
	52	Female	709 (328)	74 (48)	93.5	0.963329	18.849912	3.306658
	54	Female	495 (345)	71 (71)	163.625	0.943434	16.293245	3.335952
	55	Male	506 (346)	55 (55)	181	0.950593	5.356277	2.443538
	57	Male	752 (331)	62 (41)	72.666667	0.972074	5.729046	2.419726
	59	Female	709 (320)	82 (42)	49.8	0.943583	9.462366	2.81217
	64	Female	509 (348)	43 (43)	151.333333	0.954813	4.277577	2.176752
	65	Female	1225 (341)	73 (53)	134.25	0.983673	12.453276	3.046082
LPR	21	Female	2525 (333)	71 (44)	72.875	0.994059	6.370635	2.485294
	23	Male	2222 (329)	111 (54)	91.5	0.985149	10.705556	3.015954
	24	Male	2180 (334)	100 (61)	88.083333	0.988532	15.660659	3.264277
	24	Female	1530 (330)	105 (58)	98.625	0.978431	15.603622	3.233754
	24	Male	1941 (341)	87 (56)	97.333333	0.990211	5.739604	2.562469
	24	Male	1093 (340)	95 (63)	94.071429	0.970723	7.309741	2.86034
	25	Male	1756 (340)	74 (46)	111	0.989749	5.001736	2.329906
	25	Male	347 (344)	38 (38)	49.142857	0.962536	5.853939	2.494268
	26	Male	12690 (334)	145 (47)	87.625	0.99803	9.110583	2.713114
	29	Female	540 (346)	52 (52)	106	0.964815	4.805943	2.493928
	30	Female	1548 (335)	84 (47)	70.333333	0.98708	5.947799	2.655301
	30	Female	641 (354)	57 (57)	131.375	0.964119	9.536172	2.812082
	31	Female	10783 (327)	154 (39)	54.111111	0.996569	6.764086	2.478413
	32	Female	12287 (325)	154 (35)	52	0.997803	6.531448	2.331705
	32	Female	462 (343)	37 (37)	152.5	0.965368	7.045405	2.410483
	32	Female	755 (328)	67 (42)	57.166667	0.966887	8.80302	2.814522
	33	Male	2214 (337)	70 (49)	60.769231	0.995032	8.543232	2.763543

	33	Male	14554 (335)	135 (34)	53.125	0.99787	5.383986	2.15124
	37	Female	21715 (330)	135 (22)	29	0.99825	3.325472	1.718054
	41	Female	4817 (327)	115 (40)	75	0.995433	5.6065	2.366512
	47	Male	775 (332)	66 (45)	79.2	0.972903	14.448067	3.027226
	50	Female	911 (329)	77 (48)	90	0.968167	8.133253	2.805591
	51	Female	949 (334)	79 (50)	119	0.969442	5.887877	2.672275
	56	Male	7926 (334)	96 (41)	71	0.997224	9.39375	2.775459
	57	Female	18301 (330)	141 (37)	60.75	0.99847	6.018293	2.267221
	57	Male	7421 (334)	139 (55)	101.5	0.997035	7.354007	2.723292
	60	Female	1141 (335)	76 (57)	80	0.983348	8.479085	2.919917
	61	Male	754 (329)	68 (43)	62	0.966844	9.588768	2.743068
**Normal**	23	Female	3952 (332)	104 (40)	73	0.993927	7.312483	2.526434
	23	Female	1810 (336)	84 (45)	66.111111	0.98895	6.84505	2.57269
	23	Female	5856 (333)	104 (33)	43.111111	0.994536	1.952596	1.45867
	24	Female	1556 (330)	86 (54)	58.4	0.985219	18.809771	3.36631
	26	Female	2121 (334)	70 (42)	55.333333	0.994342	9.313515	2.761327
	28	Male	7509 (328)	128 (45)	77.5	0.996271	5.820905	2.40857
	37	Male	808 (336)	60 (44)	61	0.980198	10.104129	2.854072
	38	Male	749 (321)	77 (40)	67.142857	0.950601	5.387601	2.439864
	40	Female	856 (327)	75 (38)	45.8	0.956776	7.445314	2.624424
	42	Female	1131 (342)	66 (47)	81.5	0.983201	9.347708	2.768241
	43	Male	746 (326)	73 (41)	68.142857	0.957105	11.07337	2.777896
	44	Female	396 (345)	55 (55)	70.3	0.954545	9.855506	3.020813
	44	Male	720 (328)	61 (31)	44.75	0.958333	7.40207	2.430376
	46	Female	860 (333)	67 (42)	50.666667	0.97093	9.442774	2.824003
	49	Female	5340 (338)	106 (42)	69.142857	0.993071	11.53829	2.827537
	50	Female	3050 (331)	95 (37)	112	0.991803	4.748305	2.040423
	50	Female	780 (334)	43 (28)	33.142857	0.980769	6.026333	2.226983
	51	Female	1624 (338)	64 (41)	72.666667	0.990148	4.344241	2.223791
	51	Male	1404 (331)	88 (48)	62.615385	0.980057	7.466165	2.67523
	51	Male	922 (336)	63 (48)	90.75	0.983731	10.447373	2.969541
	52	Female	11038 (338)	133 (40)	53.909091	0.997826	7.077232	2.452329
	52	Male	1621 (331)	69 (41)	81.625	0.986428	4.150018	2.038909
	52	Female	747 (336)	60 (35)	56	0.966533	5.328355	2.313864
	53	Male	4879 (334)	99 (31)	86	0.991392	10.252765	2.692441
	55	Male	415 (344)	57 (57)	69.65	0.944578	8.568773	2.861201
	56	Female	5249 (331)	93 (42)	59	0.996952	7.142951	2.59359
	56	Male	3494 (334)	93 (47)	70.333333	0.994848	10.949203	2.892868
	58	Male	531 (345)	51 (51)	152.5	0.960452	9.177235	2.759552
	59	Male	3148 (332)	88 (36)	61.5	0.993329	4.091898	2.101202
	65	Female	484 (344)	58 (58)	135.5	0.958678	9.074912	2.940564

Cohort	Age	Gender	# of sequences (normalized)	Observed # of species (normalized)	Chao 1 (richness)	Good's coverage	1/Simpson (diversity)	Shannon (diversity)
**Smoker**								
**GERD**	25	Male	1205 (339)	79 (59)	152	0.976763	12.825386	3.100289

	26	Male	341 (341)	46 (46)	65.428571	0.950147	11.178172	2.981881
	42	Female	1202 (335)	114 (69)	177.111111	0.960067	13.871808	3.254076
	49	Male	8580 (334)	120 (46)	75.666667	0.997319	5.583434	2.434524
	49	Female	3167 (336)	93 (34)	61.25	0.988633	2.502891	1.624609
	50	Female	2741 (328)	67 (20)	36.5	0.98942	1.277709	0.656768
	50	Female	861 (329)	52 (30)	41.25	0.969803	5.160784	2.285615
	52	Female	2558 (335)	145 (72)	158.666667	0.982799	13.958333	3.330632
	54	Male	949 (330)	79 (51)	88.333333	0.967334	12.03125	3.061717
	61	Female	1366(329)	41 (23)	45	0.986823	2.115009	1.29689
**LPR**	26	Female	1760 (338)	63 (39)	60.857143	0.995455	4.527266	2.197808
	27	Male	2540 (334)	78 (36)	58.666667	0.987795	6.117149	2.338845
	29	Male	5514 (332)	133 (57)	93.142857	0.993653	14.734781	3.260401
	34	Female	4393 (336)	70 (23)	29	0.994081	3.011397	1.666105
	37	Female	2202 (336)	70 (39)	56	0.993188	5.891343	2.317672
	49	Male	1799 (331)	96 (42)	55.153846	0.97721	3.842068	2.189605
**Normal**	32	Male	1474 (335)	46 (27)	42	0.99118	3.61612	1.915563
	34	Male	1495 (329)	101 (51)	84	0.976589	10.057036	2.926353
	48	Female	917 (337)	29 (17)	35.333333	0.986914	1.166619	0.458877
	60	Female	716 (338)	23 (20)	23	0.99581	2.114225	1.380005

*Following calculation of Good's coverage, all sequences were normalized to 340 sequences using the following calculation: (n sequences of OTU in x sample/total n sequences in x sample) * 340. Due to rounding to a whole number, the summed number of normalized sequences in each sample is variable. All downstream analyses were performed on normalized data.

Table S2. OTU Counts and Classifications (see .xlsx file)

Table S3. ANOVA and Tukey Testing of Diversity Metrics

**ANOVA**	chao	shannon	invsimpson
smoke	0.252	**0.002**	0.088
reflux	**0.002**	0.131	0.077
age	0.686	0.154	0.228
sex	0.945	0.131	0.444
random	0.503	0.569	0.717
smoke*reflux	0.847	0.238	0.534

**TukeyHSD**			
smoker vs nonsmoker	0.250	**0.002**	0.089
LPR vs GERD	0.065	0.607	0.161
Norm vs GERD	**0.002**	0.123	0.105
Norm vs LRP	0.423	0.537	0.974

**TukeyHSD**			
Smoker:Normal vs NonSmoker:GERD	0.054	**0.005**	0.081
Smoker:Normal vs NonSmoker:LPR	0.385	**0.016**	0.451
Smoker:Normal vs NonSmoker:Normal	0.734	**0.031**	0.403
Smoker:GERD vs NonSmoker:GERD	0.949	0.401	0.767
NonSmoker:LPR vs NonSmoker:GERD	0.443	0.954	0.506
Smoker:LPR vs NonSmoker:GERD	0.186	0.434	0.492
NonSmoker:Normal vs NonSmoker:GERD	0.054	0.780	0.573
NonSmoker:LPR vs Smoker:GERD	0.994	0.766	1
Smoker:LPR vs Smoker:GERD	0.690	1	0.991
NonSmoker:Normal vs Smoker:GERD	0.744	0.918	1
Smoker:Normal vs Smoker:GERD	0.299	0.266	0.566
Smoker:LPR vs NonSmoker:LPR	0.823	0.747	0.983
NonSmoker:Normal vs NonSmoker:LPR	0.865	0.997	1
NonSmoker:Normal vs Smoker:LPR	0.993	0.884	0.971
Smoker:Normal vs Smoker:LPR	0.975	0.476	0.903

Table S4. PERMANOVA Analyses (p-values) of Bray-Curtis and Jaccard Indices

	BRAY-CURTIS	JACCARD
**SMOKE**	**0.028**	**0.026**
**REFLUX**	0.162	0.171
**AGE**	0.544	0.672
**SEX**	0.563	0.608
**RANDOM**	0.125	0.151
**SMOKE*REFLUX**	0.714	0.727

Table S5. SIMPER Analysis of PERMANOVA

SMOKE	%*	PHYLUM	CLASS	ORDER	FAMILY	GENUS
**OTU1**	17.6	Firmicutes	Bacilli	Lactobacillales	Streptococcaceae	*Streptococcus*
**OTU2**	11.0	Proteobacteria	Betaproteobacteria	Burkholderiales	Comamonadaceae	unclassified
**OTU3**	6.81	Bacteroidetes	Flavobacteriia	Flavobacteriales	Weeksellaceae	*Cloacibacterium*
**OTU4**	4.95	Proteobacteria	Epsilonproteobacteria	Campylobacterales	Helicobacteraceae	*Helicobacter*
**OTU6**	3.71	Bacteroidetes	Bacteroidia	Bacteroidales	Prevotellaceae	*Prevotella*
**OTU9**	3.25	Firmicutes	Bacilli	Lactobacillales	Streptococcaceae	*Streptococcus*
**OTU10**	3.05	Actinobacteria	Actinobacteria	Actinomycetales	Propionibacteriaceae	*Propionibacterium*
**OTU28**	2.69	Firmicutes	Bacilli	Bacillales	Paenibacillaceae	*Paenibacillus*
**OTU35**	2.58	Proteobacteria	Betaproteobacteria	Burkholderiales	Comamonadaceae	unclassified
**OTU8**	2.47	Firmicutes	Clostridia	Clostridiales	Veillonellaceae	unclassified
**OTU34**	2.17	Proteobacteria	Gammaproteobacteria	Pseudomonadales	Moraxellaceae	*Moraxella*
**OTU26**	1.73	Firmicutes	Bacilli	Bacillales	Staphylococcaceae	*Staphylococcus*
**OTU11**	1.63	Proteobacteria	Gammaproteobacteria	Pseudomonadales	Moraxellaceae	*Acinetobacter*
**OTU12**	1.53	Firmicutes	Bacilli	Bacillales	unclassified	unclassified
**OTU15**	1.45	Proteobacteria	Betaproteobacteria	Rhodocyclales	Rhodocyclaceae	*Dechloromonas*
**OTU14**	1.44	Proteobacteria	Gammaproteobacteria	Pseudomonadales	Pseudomonadaceae	*Pseudomonas*
**OTU21**	1.33	Proteobacteria	Betaproteobacteria	Burkholderiales	Comamonadaceae	*Aquabacterium*
**OTU13**	1.21	Bacteroidetes	Bacteroidia	Bacteroidales	Paraprevotellaceae	*Prevotella*

*Percent contribution of differences seen in PERMANOVA related to smoking.

Table S6. PICRUSt Analysis (see .xlsx file)

Table S7. ANOVA* of Richness and Diversity Data Comparing False Vocal Fold Biopsies and Vocal Fold Lesions.

ANOVA	CHAO	SHANNON	INVSIMPSON
**TYPE FVF**-ALL LESIONS**	**1.29E-09**	**4.43E-12**	**0.001**
**ANOVA**	chao	shannon	invsimpson
**TYPEALL**	**2.20E-07**	**3.21E-10**	**0.006**
**TUKEYHSD**			
**FVF VS CYST**	0.114	0.178	0.999
**NODULE VS CYST**	0.989	0.965	0.864
**POLYP VS CYST**	0.992	0.625	0.624
**REINKE VS CYST**	0.954	0.591	0.559
**NODULE VS FVF**	0.061	0.054	0.588
**POLYP VS FVF**	**1.66E-04**	**4.00E-07**	**0.043**
**REINKE VS FVF**	**1.56E-04**	**2.90E-06**	**0.048**
**POLYP VS NODULE**	1.000	0.989	1.000
**REINKE VS NODULE**	1.000	0.981	0.999
**REINKE VS POLYP**	0.996	1.000	1.000

*TukeyHSD for multiple comparisons
**FVF=False Vocal Fold

Table S8. PERMANOVA to Compare False Vocal Fold Biopsies and Vocal Fold Lesions

Type: FVF* vs lesion

	BRAY-CURTIS	JACCARD
**TYPE FVF-ALL LESIONS**	**1.00E-04**	**1.00E-04**
**RANDOM**	0.914	0.883

TypeAll: FVF vs Reinke vs cyst vs polyp vs nodule

	BRAY-CURTIS	JACCARD
**TYPE FVF-LESION TYPES**	**1.00E-04**	**1.00E-04**
**RANDOM**	0.875	0.830

Pairwise PERMANOVA between all groups, Bonferroni correction for multiple comparisons

	BRAY-CURTIS	JACCARD
**FVF VS REINKE**	**0.01**	**0.01**
**FVF VS CYST**	**0.01**	**0.01**
**FVF VS NODULE**	**0.03**	0.15
**FVF VS POLYP**	**0.01**	**0.01**
**REINKE VS CYST**	1	1
**REINKE VS NODULE**	1	1
**REINKE VS POLYP**	1	1
**CYST VS NODULE**	1	1
**CYST VS POLYP**	1	1
**NODULE VS POLYP**	1	1

*FVF=False Vocal Fold
